# NADH Dehydrogenase Subunit-2 237 Leu/Met Polymorphism Modulates the Effects of Coffee Consumption on the Risk of Hypertension in Middle-Aged Japanese Men

**DOI:** 10.2188/jea.JE20081040

**Published:** 2009-09-05

**Authors:** Akatsuki Kokaze, Mamoru Ishikawa, Naomi Matsunaga, Kanae Karita, Masao Yoshida, Tadahiro Ohtsu, Takako Shirasawa, Hideaki Sekii, Taku Ito, Teruyoshi Kawamoto, Yutaka Takashima

**Affiliations:** 1 Department of Public Health, Showa University School of Medicine, Tokyo, Japan; 2 Department of Public Health, Kyorin University School of Medicine, Tokyo, Japan; 3 Mito Red Cross Hospital, Mito-shi, Ibaraki, Japan

**Keywords:** coffee consumption, hypertension, NADH dehydrogenase, polymorphism, personalized preventive medicine

## Abstract

**Background:**

Habitual coffee consumption has been reported to lower blood pressure in the Japanese population. The NADH dehydrogenase subunit-2 237 leucine/methionine (ND2-237 Leu/Met) polymorphism is associated with longevity and modifies the effects of alcohol consumption on blood pressure in the Japanese population. The objective of this study was to determine whether this polymorphism also modifies the effects of coffee consumption on blood pressure or the risk of hypertension in middle-aged Japanese men.

**Methods:**

A total of 398 men (mean age ± standard deviation, 53.8 ± 7.8 years) were selected from among individuals visiting the hospital for regular medical check-ups. Hypertension was defined as a systolic blood pressure ≥140 mm Hg, diastolic blood pressure ≥90 mm Hg, or antihypertensive drug treatment. Polymerase chain reaction-restriction fragment length polymorphism using the restriction enzyme *Alu*I was performed to determine ND2-237 Leu/Met genotype.

**Results:**

In subjects with ND2-237Leu, coffee consumption was significantly and negatively associated with diastolic blood pressure (*P* = 0.007). The odds ratio (OR) for hypertension was significantly lower in subjects with ND2-237Leu who consumed 2 or 3 cups of coffee per day than in those who consumed less than 1 cup of coffee per day (OR, 0.517; 95% confidence interval [CI], 0.276 to 0.968; *P* = 0.039). After adjustment, the OR remained significant (OR = 0.399; 95% CI, 0.184 to 0.869; *P* = 0.020). Moreover, after adjustment, the OR was significantly lower in subjects with ND2-237Leu who consumed more than 4 cups of coffee per day than in those who consumed less than 1 cup of coffee per day (OR, 0.246; 95% CI, 0.062 to 0.975; *P* = 0.046). However, the association between ND2-237Met genotype and hypertension did not depend on coffee consumption.

**Conclusions:**

The present results suggest that the ND2-237 Leu/Met polymorphism modulates the effects of coffee consumption on hypertension risk in middle-aged Japanese men.

## INTRODUCTION

Coffee is one of the most popular beverages in Japan. Coffee consumption was recently reported to reduce mortality from hepatocellular carcinoma^1^; to decrease the incidences of type 2 diabetes,^2^ impaired glucose tolerance,^3^ metabolic syndrome,^4^ and constipation^5^; and to lower serum uric acid concentration^6^ in the Japanese population. A large-scale epidemiological study also reported that habitual coffee consumption is associated with lower blood pressure in the Japanese population.^7^

The mitochondrial DNA cytosine/adenine (Mt5178 C/A) polymorphism,^8^ which is also known as the NADH dehydrogenase subunit-2 237 leucine/methionine (ND2-237 Leu/Met) polymorphism, is a mitochondrial DNA polymorphism that is associated with longevity.^8^^–^^13^ Among Japanese, the frequency of the ND2-237Met (Mt5178A) genotype is significantly higher in centenarians than in the general population.^8^ This polymorphism is associated with serum lipid levels,^14^ fasting plasma glucose levels,^15^ pulmonary function,^16^ intraocular pressure,^17^ and serum electrolyte levels.^18^ Moreover, the ND2-237 Leu/Met polymorphism modifies the effects of alcohol consumption on blood pressure,^19^ the risk of hypertension,^20^ serum lipid levels,^21^ serum uric acid levels,^22^ and intraocular pressure.^17^ It also modifies the effects of habitual smoking on serum lipid levels,^21^ hematological parameters,^23^ pulmonary function,^16^ intraocular pressure,^17^ and serum protein fraction levels.^24^ As compared to Japanese with ND2-237Leu, those with ND2-237Met are more resistant to lifestyle-related adult-onset diseases, such as hypertension,^20^ diabetes,^25^ myocardial infarction,^26^^,^^27^ and cerebrovascular disorders.^28^ Primary prevention of hypertension is crucial for the prevention of adult-onset atherosclerotic diseases. Therefore, differences in the blood pressure-lowering effects of coffee consumption between the ND2-237Leu and ND2-237Met genotypes are of clinical interest.

The objective of this study was to investigate whether the ND2-237 Leu/Met polymorphism modifies the effects of coffee consumption on blood pressure or the risk of hypertension in middle-aged Japanese men.

## METHODS

### Subjects

Participants were recruited from among individuals visiting the Mito Red Cross Hospital for regular medical check-ups from August 1999 through August 2000. This study was conducted in accordance with the Declaration of Helsinki and was approved by the Ethics Committee of the Kyorin University School of Medicine. Written informed consent was obtained from 602 volunteers before participation. Women were excluded because the number of women was insufficient for classification into groups based on ND2-237 Leu/Met genotype and coffee consumption. Thus, 406 men without diabetes were enrolled in the study. Patients with diabetes were excluded because the prevalence of hypertension is higher in diabetic patients than in non-diabetic patients.^29^ Eight individuals with ambiguous data were also excluded. Therefore, the subjects comprised 398 Japanese men (mean age ± standard deviation [SD], 53.8 ± 7.8 years).

### Clinical characteristics of subjects

Determination of blood chemical and physical data was conducted as described previously.^14^^,^^19^ For both systolic blood pressure (SBP) and diastolic blood pressure (DBP), the mean of 2 consecutive values, measured by physicians, was used. Hypertension was defined as an SBP of 140 mm Hg or higher, a DBP of 90 mm Hg or higher, or antihypertensive drug treatment. Body-mass index (BMI) was defined as the ratio of subject weight (kg) to the square of subject height (m). Data on coffee intake, green tea intake, frequency of alcohol consumption, and smoking status were collected by means of a questionnaire. Coffee consumption was classified by number of cups of coffee per day (1 cup or less per day, 2 or 3 cups per day, and 4 or more cups per day). Alcohol consumption was classified by drinking frequency (daily drinker; occasional drinker, which included those who drink several times per week or per month; and non- or ex-drinker). Smoking status was classified as non- or ex-smoker and current smoker.

### Genotyping

Genotyping was performed as described previously.^14^ Briefly, DNA was extracted from white blood cells. Polymerase chain reaction-restriction fragment length polymorphism using the restriction enzyme *Alu*I was performed. The absence of an *Alu*I site was designated as ND2-237Met (Mt5178A), and the presence of this restriction site was designated as ND2-237Leu (Mt5178C).

### Statistical analyses

Statistical analyses were performed using SAS statistical software, version 8.2 for Windows (SAS Institute, Inc., Cary, NC, 1999). For multiple linear regression analysis, frequency of alcohol consumption (non- or ex-drinkers = 0, occasional drinkers = 1, and daily drinkers = 2), smoking status (non- or ex-smokers = 0, current smokers = 1), and antihypertensive treatment (no drug treatment = 0, taking medicine = 1) were numerically coded. Multiple logistic regression analysis was used to calculate odds ratios for hypertension. A *P* value less than 0.05 was considered to be statistically significant.

## RESULTS

Except for information on coffee and green tea consumption, the clinical characteristics of study subjects were as shown previously.^20^ No significant differences in biophysical or biochemical characteristics were observed between individuals with the ND2-237Leu and ND2-237Met genotypes (Table 1). The frequency of hypertension was significantly higher in men with the ND2-237Leu genotype than in those with the ND2-237Met genotype (*P* = 0.040). No significant difference in coffee consumption was observed between the ND2-237 Leu/Met genotypes.

**Table 1.** Clinical characteristics of study subjects, by ND2-237 Leu/Met genotype

	ND2-237Leu	ND2-237Met	*P* value
	*n* = 242	*n* = 156	
Age (y)	54.4 ± 7.8	53.2 ± 7.8	0.137
Body-mass index (kg/m^2^)	23.3 ± 2.8	23.5 ± 2.6	0.452
Systolic blood pressure (mm Hg)	125.8 ± 15.7	125.8 ± 14.1	0.977
Diastolic blood pressure (mm Hg)	74.0 ± 10.5	73.8 ± 9.1	0.854
Total cholesterol (mg/dl)	203.2 ± 33.9	201.9 ± 31.9	0.699
High-density lipoprotein cholesterol (mg/dl)	54.4 ± 13.5	56.3 ± 16.1	0.184
Fasting plasma glucose (mg/dl)	99.6 ± 16.2	98.2 ± 20.2	0.399
Uric acid (mg/dl)	5.92 ± 1.23	5.94 ± 1.22	0.871
Coffee consumption (≤1 cup per day/2–3 cups per day/≥4 cups per day) (%)	60.7/30.2/9.1	54.5/33.3/12.2	0.402
Green tea consumption (≤3 cups per day/4–9 cups per day/≥10 cups per day) (%)	56.6/33.1/10.3	61.5/29.5/9.0	0.621
Frequency of alcohol consumption (daily/occasional/non- or ex-drinker) (%)	46.3/35.1/18.6	48.7/37.8/13.5	0.403
Current smokers (%)	41.7	41.0	0.888
Antihypertensive medication (%)	19.8	13.5	0.101
Hypertension (%)	32.6	23.1	0.040

Age, body-mass index, systolic blood pressure, diastolic blood pressure, serum total cholesterol level, serum high-density lipoprotein cholesterol level, fasting plasma glucose level, and serum uric acid level are shown as means ± S.D. All *P* values depict the significance of differences between individuals with the ND2-237Leu or ND2-237Met genotype. For coffee consumption, green tea consumption, alcohol consumption, smoking status, use of antihypertensive medication, and hypertension, *P *values were calculated by the chi-square test.

On analysis of variance, no interaction between ND2-237 Leu/Met polymorphism and coffee consumption on SBP or DBP was observed (*P* = 0.171 and *P* = 0.189, respectively).

On multiple linear regression analysis (Table 2), in subjects with ND2-237Leu, age, BMI, frequency of alcohol consumption, and use of antihypertensive drugs were significantly and positively associated with SBP (*P* = 0.041, *P* < 0.001, *P* < 0.001, and *P* = 0.002, respectively); BMI, frequency of alcohol consumption, and use of antihypertensive drugs were significantly and positively associated with DBP (*P* < 0.001, *P* < 0.001, and *P* < 0.001, respectively). Coffee consumption (number of cups per day) was significantly and negatively associated with DBP (*P* = 0.007). In subjects with ND2-237Met, age and use of antihypertensive drugs were significantly and positively associated with SBP (*P* = 0.015 and *P* < 0.001, respectively); BMI and use of antihypertensive drugs were significantly and positively associated with DBP (*P* = 0.001 and *P* = 0.002, respectively).

**Table 2.** Multiple linear regression analyses for blood pressure in men with the ND2-237Leu and ND2-237Met genotypes

	ND2-237Leu			ND2-237Met		
		
	Partial regression coefficient	SEM	*P* value	Partial regression coefficient	SEM	*P* value
Systolic blood pressure						
​ Age (y)	0.263	0.128	0.041	0.347	0.141	0.015
​ Body-mass index (kg/m^2^)	1.797	0.360	<0.001	0.681	0.423	0.110
​ Total cholesterol (mg/dl)	0.013	0.027	0.630	0.008	0.032	0.807
​ HDL-cholesterol (mg/dl)	0.133	0.074	0.074	−0.049	0.068	0.471
​ Fasting plasma glucose (mg/dl)	0.033	0.050	0.513	0.157	0.094	0.095
​ Uric acid (mg/dl)	−0.106	0.763	0.890	0.747	0.828	0.368
​ Coffee consumption (cups/day)	−1.049	0.646	0.106	−0.816	0.643	0.207
​ Green tea consumption (cups/day)	−0.333	0.309	0.283	−0.301	0.333	0.367
​ Frequency of alcohol consumption	4.311	1.274	<0.001	−0.395	1.421	0.782
​ Smoking status	−1.088	1.905	0.568	−2.656	2.082	0.204
​ Use of antihypertensives	7.489	2.389	0.002	15.299	2.954	<0.001
	*R*^2^ = 0.271		<0.001	*R*^2^ = 0.334		<0.001

Diastolic blood pressure						
​ Age (y)	0.006	0.085	0.939	0.144	0.098	0.145
​ Body-mass index (kg/m^2^)	1.237	0.239	<0.001	0.958	0.294	0.001
​ Total cholesterol (mg/dl)	0.008	0.018	0.633	−0.001	0.022	0.964
​ HDL-cholesterol (mg/dl)	0.016	0.049	0.754	0.006	0.047	0.906
​ Fasting plasma glucose (mg/dl)	0.005	0.033	0.889	0.032	0.065	0.628
​ Uric acid (mg/dl)	0.364	0.507	0.474	0.577	0.575	0.318
​ Coffee consumption (cups/day)	−1.163	0.430	0.007	−0.141	0.447	0.753
​ Green tea consumption (cups/day)	−0.176	0.205	0.392	−0.264	0.231	0.255
​ Frequency of alcohol consumption	2.854	0.848	<0.001	−0.760	0.987	0.443
​ Smoking status	−1.547	1.267	0.223	−1.358	1.446	0.349
​ Use of antihypertensives	5.349	1.589	<0.001	6.594	2.052	0.002
	*R*^2^ = 0.282		<0.001	*R*^2^ = 0.226		<0.001

For multiple linear regression analysis, some independent variables were numerically coded: frequency of alcohol consumption (non-/ex-alcohol drinker = 0, occasional drinker = 1, daily drinker = 2), smoking status (non-/ex-smoker = 0, current smoker = 1), and use of antihypertensives (no = 0, current = 1).

Abbreviations: HDL, high-density lipoprotein; SEM, standard error of the mean.

On multiple logistic regression analysis (Table 3), associations between the ND2-237 Leu/Met polymorphism and hypertension depended on coffee consumption. Among all subjects, the odds ratio (OR) for hypertension was significantly lower in those who consumed 2 or 3 cups of coffee per day than in those who consumed 1 cup or less per day (OR, 0.490; 95% CI, 0.295 to 0.815; *P* = 0.008). However, after adjustment, this association between coffee consumption and hypertension disappeared. The OR for hypertension was significantly lower in subjects with ND2-237Leu who consumed 2 or 3 cups of coffee per day than in those who consumed 1 cup or less per day (OR = 0.517, 95% CI: 0.276 to 0.968, *P* = 0.039). After adjustment for age, BMI, frequency of alcohol consumption, smoking status, serum total cholesterol level, serum HDL-cholesterol level, fasting plasma glucose level, serum uric acid level, and green tea consumption (number of cups per day), the OR remained significant (OR, 0.399; 95% CI, 0.184 to 0.869; *P* = 0.020). Moreover, after adjustment, the OR for hypertension was significantly lower in subjects with ND2-237Leu who consumed 4 or more cups of coffee per day than in those who consumed 1 cup or less per day (OR, 0.246; 95% CI, 0.062 to 0.975; *P* = 0.046). However, the association between the ND2-237Met genotype and hypertension did not depend on coffee consumption.

**Table 3.** Odds ratios (ORs) and 95% confidence intervals (CIs) for hypertension, by ND2-237 Leu/Met genotype and coffee consumption

Genotype and coffee consumption	Frequency		OR (95% CI)	Adjusted OR^a^ (95% CI)	Adjusted OR^b^ (95% CI)

	Normotensive	Hypertensive			
Total					
​ ≤1 cup per day (%)	151 (65.1)	81 (34.9)	1 (reference)	1 (reference)	1 (reference)
​ 2–3 cups per day (%)	99 (79.2)	26 (20.8)	0.490 (0.295–0.815)^d^	0.595 (0.352–1.028)	0.560 (0.342–1.010)
​ ≥4 cups per day (%)	33 (80.5)	8 (19.5)	0.452 (0.199–1.020)	0.580 (0.235–1.415)	0.581 (0.231–1.447)
					
ND2-237Leu					
​ ≤1 cup per day (%)	90 (61.2)	57 (38.8)	1 (reference)	1 (reference)	1 (reference)
​ 2–3 cups per day (%)	55 (75.3)	18 (24.7)	0.517 (0.276–0.968)^c^	0.527 (0.258–1.078)	0.399 (0.184–0.869)^c^
​ ≥4 cups per day (%)	18 (81.8)	4 (18.2)	0.351 (0.113–1.090)	0.333 (0.092–1.206)	0.246 (0.062–0.975)^c^
					
ND2-237Met					
​ ≤1 cup per day (%)	61 (71.8)	24 (28.2)	1 (reference)	1 (reference)	1 (reference)
​ 2–3 cups per day (%)	44 (84.6)	8 (15.4)	0.462 (0.190–1.124)	0.782 (0.287–2.131)	0.695 (0.244–1.983)
​ ≥4 cups per day (%)	15 (79.0)	4 (21.0)	0.678 (0.204–2.250)	1.373 (0.350–5.382)	1.587 (0.388–6.495)

^a^OR adjusted for age, body-mass index, alcohol consumption, and smoking status.

^b^OR adjusted for age, body-mass index, frequency of alcohol consumption, smoking status, serum total cholesterol level, serum high-density lipoprotein cholesterol level, fasting plasma glucose level, serum uric acid level, and green tea consumption.

^c^*P* < 0.05, ^d^*P* < 0.01.

## DISCUSSION

In the present study, we found that the ND2-237 Leu/Met polymorphism modulates the effects of coffee consumption on the risk of hypertension in middle-aged Japanese men. Coffee consumption was significantly and negatively associated with DBP only in men with ND2-237Leu. For men with the ND2-237Leu genotype, habitual coffee drinking may reduce the risk of hypertension.

Individuals with the ND2-237Met genotype may be more resistant to atherosclerosis than those with the ND2-237Leu genotype.^26^^–^^28^^,^^30^ The antiatherogenic advantages of the ND2-237Met genotype may be mediated by the biophysical and biochemical properties of ND2-237Met. Methionine residues play a role as an antioxidant that scavenges reactive oxygen species (ROS).^31^ NADH dehydrogenase is involved in the production of ROS^32^ and is a target of ROS. Habitual alcohol consumption influences the production of ROS by NADH dehydrogenase^33^ and modulates the susceptibility to ROS of mitochondrial proteins, including NADH dehydrogenase.^34^ Smoking attenuates the activity of NADH dehydrognase.^35^ We hypothesized that ND2-237 Leu/Met may result in differences in biophysical or biochemical status, thereby affecting ROS production and/or sensitivity related to ethanol intake or smoking. Moreover, because ROS are pathophysiologically involved with hypertension, atherosclerosis, and aging,^36^ the protective potential of ND2-237Met against ROS may play an important role in resistance to atherosclerotic diseases and increased longevity.

Chlorogenic acids, which are present in coffee beans, are assumed to be involved in the mechanism by which the ND2-237 Leu/Met polymorphism modulates the effects of coffee intake on the risk of hypertension. Pavlica and Gebhardt reported that chlorogenic acids protected against ROS in differentiated neuronal PC12 cells.^37^ Suzuki et al reported that chlorogenic acids inhibit excessive ROS production and improve hypertension in spontaneously hypertensive rats.^38^ Results from randomized clinical trials suggest that chlorogenic acids decrease blood pressure in mildly hypertensive patients.^39^^,^^40^ To elucidate the mechanisms responsible for differences between the ND2-237 Leu/Met genotypes in the blood pressure-lowering effects of chlorogenic acids, further biophysical and biochemical studies are required. Moreover, because other polymorphisms may be associated with hypertension via oxidative stress,^41^^,^^42^ investigation of gene–gene and gene–gene–environment interactions of ROS with hypertension is required.

Reports have suggested that the ND2-237 Leu/Met polymorphism modifies the effects of habitual alcohol consumption on blood pressure^19^ and the risk of hypertension.^20^ Therefore, to reduce the risk of hypertension, men with ND2-237Leu should avoid daily alcohol consumption and drink 2 or more cups of coffee per day. Clinical experimental studies have shown that drinking more than 3 cups of coffee per day lowers blood pressure in hypertensive or prehypertensive men who consume alcohol every day.^43^ Moderate coffee consumption may also lower the risk of coronary heart disease.^44^ However, heavy coffee consumption does not appear to be beneficial to overall health. A large cohort study found that an intake of more than 4 cups of coffee per day increased the risk of pancreatic cancer in Japanese men.^45^

A potential weakness of the present study was the small sample size. If there were an interaction between ND2-237 Leu/Met polymorphism and coffee consumption on blood pressure, it would be statistically difficult to detect with the present sample size. Moreover, we analyzed only a single population. To avoid chance errors in molecular epidemiological studies, it is necessary to analyze 2 or more independent data sets. Therefore, further collaborative research using a larger study sample that includes multiple populations is required. In addition to the absence of information on sodium intake and volume of alcohol consumed, coffee consumption was ambiguously defined as number of cups consumed per day. The possibility of an interaction between the ND2-237 Leu/Met polymorphism and the amount of chlorogenic acids, caffeine, or other compounds in coffee and the risk of hypertension warrants further investigation.

In conclusion, the ND2-237 Leu/Met polymorphism may modulate the effects of habitual coffee consumption on the risk of hypertension in middle-aged Japanese men. For men with ND2-237Leu, drinking 2 or more cups of coffee per day may reduce the risk of hypertension, presumably by lowering DBP. Therefore, although individuals with ND2-237Leu may have a higher risk of hypertension than those with ND2-237Met, lifestyle habits may reduce the risks of hypertension and subsequent atherosclerotic diseases. We believe that these findings may encourage further investigation of gene–environment and gene–gene–environment interactions involving habitual coffee intake on hypertension, and may contribute to the development of individualized prevention strategies for hypertension, which will result in a lower incidence of atherosclerotic diseases.

## References

[1] Kurozawa Y , Ogimoto I , Shibata A , Nose T , Yoshimura T , Suzuki H , Coffee and risk of death from hepatocellular carcinoma in a large cohort study in Japan . Br J Cancer. 2005;93:607–10 16091758 10.1038/sj.bjc.6602737 PMC2361599

[2] Iso H , Date C , Wakai K , Fukui M , Tamakoshi A ; JACC Study Group. The relationship between green tea and total caffeine intake and risk for self-reported type 2 diabetes among Japanese adults . Ann Intern Med. 2006;144:554–62 16618952 10.7326/0003-4819-144-8-200604180-00005

[3] Yamaji T , Mizoue T , Tabara S , Ogawa S , Yamaguchi K , Shimizu E , Coffee consumption and glucose tolerance status in middle-aged Japanese men . Diabetologia. 2004;47:2145–51 15662555 10.1007/s00125-004-1590-5

[4] Hino A , Adachi H , Enomoto M , Furuki K , Shigetoh Y , Otsuka M , Habitual coffee but not green tea consumption in inversely associated with metabolic syndrome. An epidemiology study in a general Japanese population . Diabetes Res Clin Pract. 2007;76:383–9 17070955 10.1016/j.diabres.2006.09.033

[5] Murakami K , Okubo H , Sasaki S Dietary intake in relation to self-reported constipation among Japanese women aged 18–20 years . Eur J Clin Nutr. 2006;60:650–7 16340942 10.1038/sj.ejcn.1602365

[6] Kiyohara C , Kono S , Honjo T , Todoroki I , Sakurai Y , Nishiwaki M , Inverse association between coffee drinking and serum uric acid concentrations in middle-aged Japanese males . Br J Nutr. 1999;82:125–30 10743484

[7] Wakabayashi K , Kono S , Shinchi K , Honjo S , Todoroki I , Sakurai Y , Habitual coffee consumption and blood pressure: A study of self-defence officials in Japan . Eur J Epidemiol. 1998;14:669–73 9849827 10.1023/a:1007478522638

[8] Tanaka M , Gong JS , Zhang J , Yoneda M , Yagi K Mitochondrial genotype associated with longevity . Lancet. 1998;351:185–6 9449878 10.1016/S0140-6736(05)78211-8

[9] Ivanova R , Lepage V , Charron D , Schächter F Mitochondrial genotype associated with French Caucasian centenarians . Gerontology. 1998;44:349 9813436 10.1159/000022041

[10] De Benedictis G , Rose G , Carrieri G , De Luca M , Falcone E , Passarino G , Mitochondrial DNA inherited variants are associated with successful aging and longevity in humans . FASEB J. 1999;13:1532–6 10463944 10.1096/fasebj.13.12.1532

[11] Ross OA , McCormack R , Curran MD , Duguid RA , Barnett YA , Rea IM , Mitochondrial DNA polymorphism: its role in longevity of the Irish population . Exp Gerontol. 2001;36:1161–78 11404057 10.1016/s0531-5565(01)00094-8

[12] Niemi AK , Moilanen JS , Tanaka M , Hervonen A , Hurme M , Lehtimaki T , A combination of three common inherited mitochondrial DNA polymorphisms promotes longevity in Finnish and Japanese subjects . Eur J Hum Genet. 2005;13:166–70 15483642 10.1038/sj.ejhg.5201308

[13] Alexe G , Fuku N , Bilal E , Ueno H , Nishigaki Y , Fujita M , Enrichment of longevity phenotype in mtDNA haplogroups D4b2b, D4a, and D5 in the Japanese population . Hum Genet. 2007;121:347–56 17308896 10.1007/s00439-007-0330-6

[14] Kokaze A , Ishikawa M , Matsunaga N , Yoshida M , Sekine Y , Teruya K , Association of the mitochondrial DNA 5178 A/C polymorphism with serum lipid levels in the Japanese population . Hum Genet. 2001;109:521–5 11735027 10.1007/s004390100602

[15] Kokaze A , Ishikawa M , Matsunaga N , Yoshida M , Makita R , Satoh M , Longevity-associated mitochondrial DNA 5178 C/A polymorphism is associated with fasting plasma glucose levels and glucose tolerance in Japanese men . Mitochondrion. 2005;5:418–25 16271520 10.1016/j.mito.2005.09.001

[16] Kokaze A , Ishikawa M , Matsunaga N , Yoshida M , Satoh M , Teruya K , Longevity-associated mitochondrial DNA 5178 C/A polymorphism and its interaction with cigarette consumption are associated with pulmonary function in middele-aged Japanese men . J Hum Genet. 2007;52:680–5 17636359 10.1007/s10038-007-0171-0

[17] Kokaze A , Yoshida M , Ishikawa M , Matsunaga N , Makita R , Satoh M , Longevity-associated mitochondrial DNA 5178 A/C polymorphism is associated with intraocular pressure in Japanese men . Clin Experiment Ophthalmol. 2004;32:131–6 15068427 10.1111/j.1442-9071.2004.00796.x

[18] Kokaze A , Ishikawa M , Matsunaga N , Yoshida M , Makita R , Satoh M , Longevity-associated NADH dehydrogenase subunit-2 polymorphism and serum electrolyte levels in middle-aged obese Japanese men . Mech Ageing Dev. 2005;126:705–9 15888325 10.1016/j.mad.2005.01.005

[19] Kokaze A , Ishikawa M , Matsunaga N , Yoshida M , Sekine Y , Sekiguchi K , Longevity-associated mitochondrial DNA 5178 A/C polymorphism and blood pressure in the Japanese population . J Hum Hypertens. 2004;18:41–5 14688809 10.1038/sj.jhh.1001632

[20] Kokaze A , Ishikawa M , Matsunaga N , Yoshida M , Satoh M , Teruya K , NADH dehydrogenase subunit-2 237 Leu/Met polymorphism modifies the effects of alcohol consumption on risk for hypertension in middle-aged Japanese men . Hypertens Res. 2007;30:213–8 17510502 10.1291/hypres.30.213

[21] Kokaze A , Ishikawa M , Matsunaga N , Yoshida M , Sekine Y , Sekiguchi K , Longevity-associated mitochondrial DNA 5178 A/C polymorphism modulates effects of daily drinking and cigarette consumption on serum triglyceride levels in middle-aged Japanese men . Exp Gerontol. 2003;38:1071–6 14580860 10.1016/s0531-5565(03)00211-0

[22] Kokaze A , Ishikawa M , Matsunaga N , Yoshida M , Satoh M , Teruya K , Longevity-associated NADH dehydrogenase subunit-2 237 Leu/Met polymorphism influences the effects of alcohol consumption on serum uric acid levels in nonobese Japanese men . J Hum Genet. 2006;51:765–71 16897192 10.1007/s10038-006-0018-0

[23] Kokaze A , Ishikawa M , Matsunaga N , Yoshida M , Makita R , Satoh M , Interaction between longevity-associated mitochondrial DNA 5178 C/A polymorphism and cigarette smoking on hematological parameters in Japanese men . Arch Gerontol Geriatr. 2005;40:113–22 15680495 10.1016/j.archger.2004.07.003

[24] Kokaze A , Ishikawa M , Matsunaga N , Yoshida M , Sekine Y , Sekiguchi K , Longevity-associated mitochondrial DNA 5178 A/C polymorphism influences effects of cigarette smoking on serum protein fraction levels in Japanese men . Mech Ageing Dev. 2003;124:765–70 12782420 10.1016/s0047-6374(03)00110-6

[25] Wang D , Taniyama M , Suzuki Y , Katagiri T , Ban Y Association of the mitochondrial DNA 5178 A/C polymorphism with maternal inheritance and onset of type 2 diabetes in Japanese patients . Exp Clin Endocrinol Diabetes. 2001;109:361–4 11573146 10.1055/s-2001-17407

[26] Mukae S , Aoki S , Itoh S , Satoh R , Nishio K , Iwata T , Mitochondrial 5178A/C genotype is associated with acute myocardial infarction . Circ J. 2003;67:16–20 12520145 10.1253/circj.67.16

[27] Takagi K , Yamada Y , Gong JS , Sone T , Yokota M , Tanaka M Association of a 5178C→A (Leu237Met) polymorphism in the mitochondrial DNA with a low prevalence of myocardial infarction in Japanese individuals . Atherosclerosis. 2004;175:281–6 15262184 10.1016/j.atherosclerosis.2004.03.008

[28] Ohkubo R , Nakagawa M , Ikeda K , Kodama T , Arimura K , Akiba S , Cerebrovascular disorders and genetic polymorphisms: mitochondrial DNA5178C is predominant in cerebrovascular disorders . J Neurol Sci. 2002;198:31–5 12039661 10.1016/s0022-510x(02)00055-2

[29] Marre M , Berrut G , Bouhanick B Hypertension and diabetes mellitus . Biomed Pharmacother. 1993;47:61–6 8218950 10.1016/0753-3322(93)90292-s

[30] Matsunaga H , Tanaka Y , Tanaka M , Gong JS , Zhang J , Nomiyama T , Antiatherogenic mitochondrial genotype in patients with type 2 diabetes . Diabetes Care. 2001;24:500–3 11289475 10.2337/diacare.24.3.500

[31] Stadtman ER , Moskovitz J , Levine RL Oxidation of methionine residues of proteins: biological consequences . Antioxid Redox Signal. 2003;5:577–82 14580313 10.1089/152308603770310239

[32] Lenaz G , Bovina C , D’Aurelio M , Fato R , Formiggini G , Genova ML , Role of mitochondria in oxidative stress and aging . Ann N Y Acad Sci. 2002;959:199–213 11976197 10.1111/j.1749-6632.2002.tb02094.x

[33] Bailey SM , Cunningham CC Contribution of mitochondria to oxidative stress associated with alcoholic liver disease . Free Radic Biol Med. 2002;32:11–6 11755312 10.1016/s0891-5849(01)00769-9

[34] Bailey SM , Pietsch EC , Cunningham CC Ethanol stimulates the production of reactive oxygen species at mitochondrial complex I and III . Free Radic Biol Med. 1999;27:891–900 10515594 10.1016/s0891-5849(99)00138-0

[35] Smith PR , Cooper JM , Govan GG , Harding AE , Scapira AH Smoking and mitochondrial function: a model for environmental toxins . Q J Med. 1993;86:657–60 8255963 10.1093/qjmed/86.10.657

[36] Valko M , Leibfritz D , Moncol J , Cronin MT , Mazur M , Telser J Free radicals and antioxidants in normal physiological functions and human disease . Int J Biochem Cell Biol. 2007;39:44–84 16978905 10.1016/j.biocel.2006.07.001

[37] Pavlica S , Gebhardt R Protective effects of ellagic and chlorogenic acids against oxidative stress in PC12 cells . Free Radic Res. 2005;39:1377–90 16298868 10.1080/09670260500197660

[38] Suzuki A , Yamamoto N , Jokura H , Yamamoto M , Fujii A , Tokumitsu I , Chlorogenic acid attenuates hypertension and improves endothelial function in spontaneously hypertensive rats . J Hypertens. 2006;24:1065–73 16685206 10.1097/01.hjh.0000226196.67052.c0

[39] Watanabe T , Arai Y , Mitsui Y , Kusaura T , Okawa W , Kajihara Y , The blood pressure-lowering effect and safety of chlorogenic acid from green coffee bean extract in essential hypertension . Clin Exp Hypertens. 2006;28:439–49 16820341 10.1080/10641960600798655

[40] Yamaguchi T , Chikama A , Mori K , Watanabe T , Shioya Y , Katsuragi Y , Hydroxyhydroquinone-free coffee: a double-blind, randomized controlled dose---response study of blood pressure . Nutr Metab Cardiovasc Dis. 2008;18:408–14 17951035 10.1016/j.numecd.2007.03.004

[41] Jiang Z , Akey JM , Shi J , Xiong M , Wang Y , Shen Y , A polymorphism in the promoter region of catalase in associated with blood pressure levels . Hum Genet. 2001;109:95–8 11479740 10.1007/s004390100553

[42] Moreno MU , José GS , Fortuño A , Beloqui Ó , Díez J , Zalba G The C242T CYBA polymorphism of NADPH oxidase is associated with essential hypertension . J Hypertens. 2006;24:1299–306 16794479 10.1097/01.hjh.0000234110.54110.56

[43] Funatsu K , Yamashita T , Nakamura H Effect of coffee intake on blood pressure in male habitual alcohol drinkers . Hypertens Res. 2005;28:521–7 16231758 10.1291/hypres.28.521

[44] Cornelis MC , El-Sohemy A Coffee, caffeine, and coronary heart disease . Curr Opin Lipidol. 2007;18:13–9 17218826 10.1097/MOL.0b013e3280127b04

[45] Lin Y , Tamakoshi A , Kawamura T , Inaba Y , Kikuchi S , Motohashi Y , Risk of pancreatic cancer in relation to alcohol drinking, coffee consumption and medical history: findings from the Japan collaborative cohort study for evaluation of cancer risk . Int J Cancer. 2002;99:742–6 12115510 10.1002/ijc.10402

